# Transcriptome and Proteome Research in Veterinary Science: What Is Possible and What Questions Can Be Asked?

**DOI:** 10.1100/2012/254962

**Published:** 2012-01-04

**Authors:** Robert Klopfleisch, Achim D. Gruber

**Affiliations:** Institut für Tierpathologie, Universität Berlin, Robert-von-Ostertag-Strasse 15, 14163 Berlin, Germany

## Abstract

In recent years several technologies for the complete analysis of the transcriptome and proteome have reached a technological level which allows their routine application as scientific tools. The principle of these methods is the identification and quantification of up to ten thousands of RNA and proteins species in a tissue, in contrast to the sequential analysis of conventional methods such as PCR and Western blotting. Due to their technical progress transcriptome and proteome analyses are becoming increasingly relevant in all fields of biological research. They are mainly used for the explorative identification of disease associated complex gene expression patterns and thereby set the stage for hypothesis-driven studies. This review gives an overview on the methods currently available for transcriptome analysis, that is, microarrays, Ref-Seq, quantitative PCR arrays and discusses their potentials and limitations. Second, the most powerful current approaches to proteome analysis are introduced, that is, 2D-gel electrophoresis, shotgun proteomics, MudPIT and the diverse technological concepts are reviewed. Finally, experimental strategies for biomarker discovery, experimental settings for the identification of prognostic gene sets and explorative versus hypothesis driven approaches for the elucidation of diseases associated genes and molecular pathways are described and their potential for studies in veterinary research is highlighted.

## 1. Background

The molecular aetiology and mechanisms of the many neoplastic, inflammatory, and degenerative veterinary diseases are largely unknown. This information deficit is caused by several constraining factors such as the lack of the complete genomic information or the unavailability of molecular tools, for instance, species-specific antibodies.

Due to this lack of a critical mass of knowledge on the disease mechanisms in veterinary medicine, hypotheses on the pathogenesis of veterinary diseases are often based on the knowledge on homologous human diseases. This approach doubtlessly helped to elucidate the aetiology of several veterinary diseases, such as the impact of mutations in the c-kit receptor for some canine mast cell tumours [1]. However, in at least the same number of studies, this approach failed to identify identical mechanism in human and animal diseases, most probably due to interspecies differences or plainly because of the absence of comparable human diseases.

An alternative approach to develop and test hypotheses is to study known cellular molecular mechanisms or genes whose dysfunction may theoretically cause the clinical symptoms and morphologic lesions. This includes the analysis of the mutational DNA status and the RNA and protein expression levels of these specific genes to elucidate their role in the disease. However, due to the redundancy of gene functions and the complexity of molecular pathways, the chances are rather low to identify “the” disease-associated genes or molecular pathways by this approach. Furthermore, it becomes more and more evident that most diseases and notably the carcinogenesis of veterinary tumours are only rarely caused by a mutation in a single gene but are rather caused by dysfunction of a wider array of multiple genes [2].

**Figure 1:**
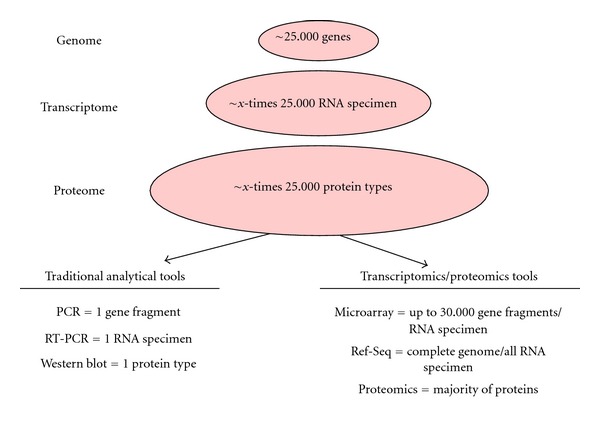
Traditional analytical tools for quantification of DNA, RNA, or proteins are restricted to the sequential analysis of one biomolecule specimen at a time. Research based on these tools is therefore strictly hypothesis driven. In contrast, transcriptomics tools permit the parallel quantification of thousands of biomolecules and therefore allowing for explorative, non-hypothesis-driven studies.

In case the above-mentioned hypothesis-driven approaches fail, non-hypothesis-driven, explorative studies are feasible alternatives. This approach, however, needs technologies which facilitate the concurrent analysis of a large scale of the genome, the transcriptome, or the proteome in diseased versus nondiseased tissues (Figure 1).

The so-called “-omics” technologies, which made much headway in the last ten years, enable such an approach to answer hitherto inaccessible scientific questions. The transcriptome [3] which constitutes the complete set of RNA specimen within a tissue, and the proteome [4] the complete set of proteins in a tissue, have been named in analogy to the genome, the complete set of genes of an organism. In addition to these three dominating fields, several other groups of biochemical elements have been coined in analogy to the genome, such as the metabolome [5], the complete set of metabolic intermediates, or the glycome [6], the complete set of cellular sugars, free or in more complex molecules.

This review aims to summarize the current state of the art of technologies in transcriptome and proteome research. Furthermore, the strengths and weaknesses of the technologies will be described, and potential applications in veterinary science will be depicted.

## 2. Methods in Transcriptomics

The term transcriptome embraces all types of complementary RNA synthesized during transcription of the genome at a given time point [3]. Of these, microRNA (miRNA) and messenger RNA (mRNA) momentarily are the RNA types of greatest interest in transcriptome research. The mRNA expression levels directly reflect the gene activity of a cell, while differences in miRNA expression levels, a major posttranscriptional regulator of gene activity, are thought to be associated with several cellular functions and diseases [7]. Three common methods for the multiplex detection and quantification of miRNA and mRNA at a large scale are momentarily used: microarrays, deep sequencing (RNA-Seq), and quantitative reverse transcription (RT) PCR arrays (Table 1).

### 2.1. Microarrays

A microarray is a multiplex, high-throughput screening method which uses a two-dimensional arrangement of nucleotide probes on a glass slide or a thin silicon chip [8]. One array can contain ten thousands of nucleotide sequences or hypothetically the complete transcriptome, which is covalently bound to the chip's surface or fixed in parallel rows in microfluidic channels [8, 9]. Depending on the scientific questions asked, these nucleotide sequences may represent short fragments (probes) with exon sequences to analyse the presence and the quantity of specific mRNA or miRNA in expression profiling experiments. Alternatively, genomic DNA sequences with allelic variants, known mutations, or single nucleotide polymorphism (SNP) is spotted on the chips to identify the mutational status of a tissue in comparative genomic analysis or SNP assays [10]. Independent from the spotted sequences, pairwise hybridization of the given sequences on the chip with complementary DNA or cDNA sequences (targets) in a tissue lysate under high-stringency conditions is the general principle of all microarray-based experiments [11]. Successful hybridization is visualized by labelling of bound target oligonucleotides with fluorescent dyes. Fluorescence intensity of the respective spot on the chips is subsequently used to determine the presence or absence of a mutation or SNP or the level of mRNA or miRNA expression in a tissue, respectively [11].

The resulting large data sets on the expression level of up to ten thousands of genes represent a challenge to the commonly used statistic methods. These challenges include the subtraction of background noise, the normalization of data to allow for the comparison of different microarrays and microarray experiments with each other, and the identification of statistically significant changes in large data sets [12]. For the latter, the common statistical test, *t*-test, ANOVA, and Mann-Whitney test have been adjusted to consider the multiple comparisons and permit cluster analysis [13, 14]. However, in most instances, the number of variables is larger than the observational units. Principal component analysis and partial least squares have been used for dimension reduction and to visualize microarray data sets [15]. Furthermore, clustering methods like hierarchical and fuzzy clustering are used to identify differences in gene expression profiles associated with different disease states or treatment response [16].

Ready-to-use whole genome cDNA microarrays are commercially available for several species including human, mouse, and rat (Affymetrix, Illumina, Agilent) but also for canine, bovine, porcine, sheep, equine, chicken, rhesus macaque, salmon, and zebrafish (Affymetrix, Agilent). However, costume-made microarrays with any given DNA of interest are provided by several local and international suppliers. These arrays can be adjusted to each experiment in terms of gene number or type of interest and may therefore represent a low-cost alternative to expensive full-genome microarrays.

Similar to other analytic tools, microarray experiments require technical and biological replicates. In addition, several initiatives are launched to standardize microarray experiments, whose outcome is strongly influenced by tissue and RNA handling, the assay protocols and the microarray platform on the resulting data. For instance, the “minimum information about a microarray experiment (MIAME)” and the “microarray quality control (MAQC) project” are becoming the gold standard of how detailed information on an experiment should be included in a publication [17, 18].

In summary, microarrays are well established and valuable tools to get a first impression on the genetic activity or the genomic composition of a specific tissue under physiologic and pathologic conditions. Despite the high quality of prefabricated microarrays, one has to keep in mind that cross-hybridization and low sensitivity when compared to quantitative PCR are intrinsic problems of this valuable method and may lead to false positive or negative results [19]. Microarray data can therefore in most circumstances not be considered as final and conclusive information but have to be confirmed by other methods such as quantitative PCR or, optimally, on the protein level or by functional cell assays [17, 18].

### 2.2. Deep Sequencing (RNA-Seq)

RNA-Seq is a new approach to obtain complete information on the RNA expression levels in a tissue sample [20]. This approach utilizes the tremendous progress in the development of next generation sequencing technologies, which allow to sequence millions of base pairs in a relatively short time [21]. Initially, these methods were used for whole-genome sequencing of organisms [22] or tumours [23]. Within the past 2-3 years, second-generation sequencing has been applied to cDNA sequencing to obtain information on the full set of all transcribed RNA sequences within a given tissue, that is, the complete transcriptome in so-called RNA-Seq experiments [24, 25].

RNA-Seq uses a cDNA library, which is reverse transcribed from the total RNA in a single tissue or cell culture [20]. Small fragments from each of these cDNA molecules are then sequenced using the different technology platforms Illumina IG [24], Applied Biosystems SOLiD [26], Roche 454 Life Science [27], or Helicos Biosciences tSMS [28].

Although subsumed under the name “next generation” or “deep” sequencing, the three main technologies are based on different principles. Pyrosequencing, which has been developed by 454 Life Sciences, is based on the emulsion amplification of a single DNA sequence attached to a primer-coated bead, on which a clonal DNA colony is formed in a picotitre well [29]. The sequencing procedure employs luciferase to generate light for indication of the addition of one of the four consecutively added dNTP types to the newly synthesised DNA in each well [29].

In contrast, the reversible dye-terminator-based technology of Ilumina employs amplification of DNA fragments to clonal DNA colonies on a glass slide. During each sequencing step, one of the four types of dNTP is added, and the addition of the fluorescently labelled nucleotides is detected by a camera at the respective spot [30]. After each step, the dye is chemically removed, and the next cycle is started.

The SOLiD technology of Applied Biosystems is based on sequencing by ligation. Similar to pyrosequencing, DNA is amplified by emulsion PCR, and the resulting bead with a clonal DNA colony is positioned on a glass slide [31, 32]. The mismatch sensitivity of the DNA ligase is then used to identify the nucleotide present at a given position in a DNA sequence.

The sequences retrieved from any of these technologies are aligned to the reference genome, and the resulting information can be used to analyze both genomic and posttranscriptional mutations and the mRNA expression level of each gene. RNA-Seq therefore extends the information obtained with microarrays by a second dimension, the sequence of the mRNA transcripts [20]. This may permit the identification of disease-associated mutations or SNP in the actively transcribed genes [26].

Another advantage of RNA-Seq is potential analysis of genomes from organisms without complete genomic sequence information, although further data analysis in these cases is often hampered by the lack of gene annotation or the lack of a sequenced genome [33]. This is in strict contrast to the microarray approach that requires knowledge on the sequences to be detected [34]. Finally, RNA-Seq also has a large dynamic range of quantification levels which allows the quantification of very low and high quantities in contrast to the low sensitivity and reduced dynamic range of microarrays [35].

A major disadvantage of this technology is the relatively high costs for a high coverage analysis of a complex mammalian genome [36]. Still, the price per nucleotide is significantly lower than for classic sequence; a reasonable RNA-Seq run requires the analysis of several million bases, making it still an expansive technology for most scientific questions.

### 2.3. Quantitative Real-Time PCR Arrays

Quantitative real-time PCR (qPCR) arrays are, although less complex, still a very valuable method for the quantification of the gene expression of up to 600 genes [37]. At the moment, qPCR arrays are a commonly used method for the quantification of the “miRNA transcriptome” [37]. This is mainly based on two facts. First, the number of miRNA types per species can be considered at level of hundreds and is therefore much lower than the number of mRNA types (see http://www.mirbase.org/, [38–40]). Second, the high similarity between the different miRNA types makes the development of microarrays difficult due to the high risk of cross-hybridization [41]. In contrast, Taq-Man- or SYBR-Green-based qPCR assays permit a specific and sensitive quantification of miRNA and are a commonly used method in veterinary sciences for the detection of mRNA and miRNA [42–46]. Arranged in an array, qPCR assays are therefore an efficient and highly reproducible method to quantify the miRNA transcriptome. 

## 3. Methods in Proteomics

Transcriptome analysis gives useful information on the transcriptional activity of a cell. However, due to posttranscriptional gene regulation and the different stabilities and biological half-lives of RNA and protein specimens including miRNA, the correlation between mRNA levels and the corresponding protein is often poor [47]. Transcriptome analysis therefore requires confirmation of the results on the protein level for most scientific questions. The analysis of the cellular proteome is, however, a much greater challenge than the transcriptome due to the high chemical diversity of the cellular proteins [48]. Despite these difficulties, proteome analysis has become an important tool for explorative analysis of molecular mechanisms in physiology and pathology [49]. There is a great variety of approaches applied in proteome research that can be grouped into two broader categories: gel-based assays and shotgun proteomics (Table 2). 

### 3.1. Gel-Based Proteomics

**Figure 2:**
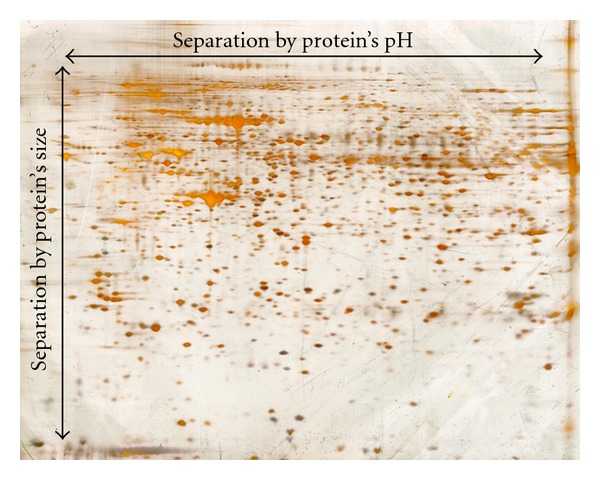
Gel-based proteomics reduces the complexity, of protein lysates by two-dimensional separation of proteins, that is, separation by size and pH. This leads to a high resolution of single protein which facilitates the quantification of their spot size and the extraction of single proteins for subsequent mass spectrometry-based identification of the protein identity.

One of the major problems of proteome analysis is the complexity of proteins in tissues and cell lysates. Two-dimensional gel electrophoresis (2D-GE) which combines two dimensions of physical protein separation is a common method to separate proteins by their chemical properties [50, 51]. In the first dimension, the proteins are separated by their isoelectric point (IP), that is, they are arranged on a linear gel with an immobilized pH-gradient according to their content of basic or acidic amino acids [51, 52]. In the second dimension, IP-separated proteins are separated by their molecular size similar to a conventional SDS-Page [51, 53]. The orthogonal combination of both techniques results in a two-dimensional arrangement of proteins with a high resolution of single protein types (Figure 2). 

The separated proteins can be visualized on the gel by different staining methods, for instance, Compassion brilliant blue [54], silver (Figure 2) [55], or fluorescent stainings [56]. Each of the detected spots on the acrylamide gel theoretically consists of one protein species, and each cell or condition of a cell is theoretically associated with a specific pattern of these spots on the gel. Changes in this spot pattern should therefore reflect changes of the cellular proteome, for example, its metabolism and gene activity under diseased or healthy condition [51].

Two approaches have been developed to compare spot presence and intensity in 2D-GE gels. In the original single channel approach, all gels containing one protein sample, are stained with same stain and scanned independently [57]. This approach is, however, prone to the technical intergel variability and has difficulties to normalize spot intensity. The multiplex approach of two-dimensional differential gel electrophoresis (2D-DIGE) uses gels which contain up to three protein samples stained with three different fluorescent dyes to reduce the gel number [58]. One of the three samples on each gel usually represents an internal standard, which allows for normalization of spot intensity between different gels [58]. 

Detection of the differences in spot intensity even between only two gels usually overstrains the human eyes capacity independent from the method used. Comparison or quantification of spot intensity and presence therefore requires automated digital image analysis [59]. Despite the high efficiency of the available software, comparison of protein expression pattern on polyacrylamide gels is still the major challenge and weakness of gel-based proteome analysis. Even with highest accuracy and long experience of the experimenter, preparation of 2D-GE gels without technical artefacts is not assured mostly due to random polymerization artefacts in nonmanufactured gels. Commercially available, machine-made gels are an interesting but costly alternative that reduces most of these gel-based artefacts [60].

Once protein spots with a significant difference in intensity between the two disease states are identified, the protein type has to be identified using mass spectrometry. To this end, the spots with the respective protein are picked and eluted from the gel and fragmented for mass spectrometry by trypsin-digestion [61]. The molecular weight of the trypsin digested protein fragments is then determined using, for instance, Matrix-assisted Laser Desorption/Ionization (MALDI) [62]. This approach uses trypsin fragment pattern, the so-called “peptide fingerprint” (PF), to identify the regulated protein in open source protein databases [63]. With this PF, protein data bases are searched to determine the peptide/protein with the highest accordance according to statistical calculations. However, PFs for animal proteomes are far from being complete, and PFs obtained from proteome studies in animals are in most cases compared with peptide masses of human protein in the versatile human data bases. This may lead to low sequence coverage [64], and peptide sequencing is required for Mascot MS/MS Ions Search (C. Weise, Freie Universität, personal communication).

In summary, gel-based proteomics is still an acceptable and commonly used approach to analyze the cellular proteome. Its main advantage is the relatively low costs for equipment when compared to non-gel-based approaches. Disadvantages are the restriction on hydrophilic proteins with a pH between 3 and 10, difficult standardization of the gel preparation, the limited dynamic range, and the limited sensitivity [65–69].

### 3.2. Shotgun Proteomics (MudPIT)

**Figure 3:**
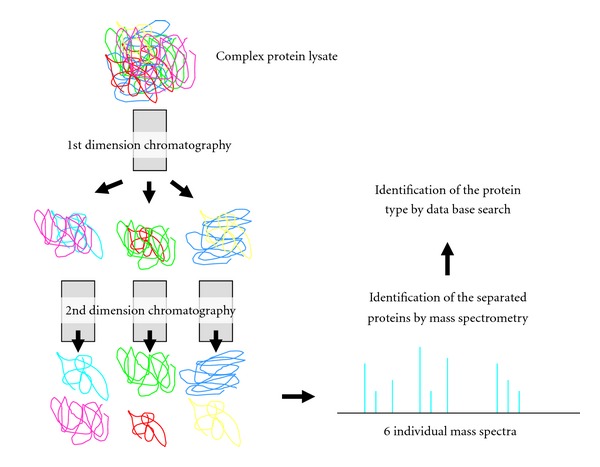
Shotgun proteomics or MudPIT uses multidimensional chromatographic separation to reduce complexity of protein lysates. The selection of the type of chromatographic columns depends on the question asked and the technical equipment. The different protein lysate fractions obtained by chromatographic separation ideally contain only one protein type which is subsequently identified by mass spectrometric analysis and data base search.

The term shotgun proteomics is applied due to its similarities with DNA shotgun sequencing, which sequences multiple short DNA fragments in complex mixtures and recombines them in silico [48]. Similarly, shotgun proteomics analyses trypsin-digested protein fragments in complex mixtures by mass spectrometry after separation by liquid chromatography. This so-called multidimensional protein identification technology (MudPIT) combines at least two chromatography separation steps with tandem mass spectrometry (Figure 3) [70, 71]. The combination of two chromatographic methods allows for the separation of digested peptide fragments by at least two features, for example, charge and hydrophobicity, to reduce the overall protein complexity [72]. The selection of two coupled, thus two-dimensional chromatography methods depends on the scientific question asked and the experience of the experimentator. Microcapillaries packed with strong cation exchange material, reverse phase material, or other affinity-based chromatographic material are most commonly used. In addition, separation before mass spectrometry may also include isoelectric focussing and capillary electrophoresis which enables a high resolution and narrow analyte bands [73]. 

The different peptide fractions are eluted from the chromatography columns by alternating salt pulses or organic mobile gradients. They are subsequently stored or, more commonly, directly applied to the mass spectrometer [71, 74]. MudPIT approaches commonly use electrospray ionization (ESI) mass spectrometers since the chromatography eluate can be directly “online” analyzed with a connected ESI mass spectrometer [75].

Most proteome experiments do not exclusively aim at identifying the general protein composition in the tissue lysates but require comparative protein quantification. Isotopic labelling or label-free methods have been developed and successfully applied in MudPIT experiments [74]. A simple method for label-free quantification is the spectral counting [76]. It uses the observation that the total number of spectra from a peptide correlates well with the original abundance of the protein in lysates [77]. However, relative quantification by spectral counting is limited to prefractionated, less complex samples [76], while analysis of complex, unfractionated samples and low abundant proteins may result in reduced accuracy [78].

Stable isotope labelling is an alternative strategy to quantify proteins in MudPIT experiments. Stable isotope labelling with amino acids in cell culture (SILAC) is a metabolic labelling strategy that has been applied to both cell cultures and small mammals [79, 80]. It relies on metabolic incorporation of “light”, native amino acids in one experimental group or “heavy” amino acids with substituted isotopic deuterium ^13^C in the other group [80]. A typical experimental design therefore includes a cell line which is grown in cell culture medium with light or heavy amino acids but under otherwise identical conditions. This leads to the complete replacement of the native amino acids in the “heavy” isotope group after a decent incubation period [81], marking the proteome of this cell unequivocal. After the intended experiment, this labelling facilitates the mixture of equal amounts of protein lysates from both experimental groups and parallel analysis by mass spectrometry, thus reducing technical artefacts during the spectrometric quantification. The different isotope mass in the amino acids of the two groups leads to twin peaks which can be quantified relatively according to their intensity ratios [81].

Isotope-coded affinity tags (ICAT) and isobaric tag for relative and absolute quantisation (iTRAQ) rely on the chemical labelling of protein samples after the experiment but before mass spectrometry [82, 83]. ICAT and iTRAQ assays label proteins with reactive groups which contain either heavy or light isotopes, which allows the direct quantification of proteins by mass spectrometry similar to SILAC [82, 83]. In contrast to ICAT and SILAC, the isobaric labelling by iTRAQ does not increase the complexity of the protein lysates and labelling of all peptides in a mixture after the biological experiment [74]. ICAT and iTRAQ therefore enable the proteome quantification in tissue samples of larger mammals which cannot be fed with substituted amino acids only.

In summary, shotgun proteomics analyses the proteome of complex protein lysates in an industrialized manner without gel-based artefacts. It permits the analysis of a wider range of proteins including hydrophobic membrane proteins and strongly acidic and basic proteins. Limitations are the restriction of this method to specialized and appropriately equipped laboratories and the relatively high costs (Table 2).

## 4. Questions to Be Asked in Transcriptomics and Proteomics

The “-omics” technologies enable the parallel analysis of several thousands of biomolecules in a sample instead of the traditional sequential approach. The comprehensive parallel approach naturally interferes with the sensitivity and specificity of detection and quantification. However, these technologies are now on a technological level that facilitates their application for explorative, descriptive pilot studies on biological phenomena when the present knowledge is not sufficient to establish a substantiated hypothesis on its molecular basis. Furthermore, they may also be applied to overcome deadlocked views and paradigms and to approach well-known diseases unbiased again.

**Figure 4:**
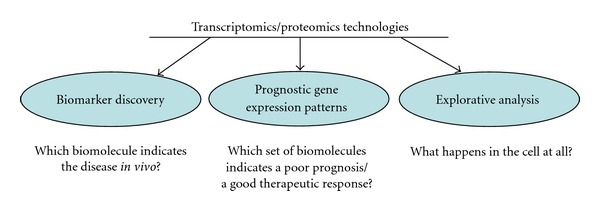
The three main fields for the application of transcriptomics and proteomics technologies and the basic questions behind these applications.

In the following, three major research fields are introduced where “-omics” technologies are successfully applied: biomarker discovery, identification of complex, prognostic gene expression patterns, explorative and hypothesis-driven proteome, and transcriptome analysis (Figure 4).

### 4.1. Biomarker Discovery

A biomarker can be DNA, mRNA, miRNA, proteins, or any other biomolecule associated with a specific state of the cell or tissue [84]. These markers are synthesized by the diseased tissue itself or by nondiseased cells in response to the neoplastic or inflammatory disease. Biomarkers are further subdivided in diagnostic marker which enable the detection of the disease, prognostic markers which in turn allow for a prediction of the disease course when the initial diagnosis has been established, and stratification markers which predict a response to a specific treatment (reviewed in [84]).

The ideal biomarker therefore permits the diagnosis and classification of a disease with a cheap, sensitive, and specific assay in a tissue sample which is obtained by a microinvasive intervention such as serum samples or small tissue biopsies.

Several, well-established biomarkers are used in clinical biochemistry in daily diagnostic routine. For instance, enzymatic activity of alkaline phosphatase (ALP) and other enzymes in serum samples are used for the evaluation of the hepatic function or thyroxin, and sex steroid levels are used to identify endocrine diseases. Tumor (bio-) markers are a field of specific interest in the search for biomarkers, and several of them have been introduced in human routine diagnostic, for instance, alpha-fetoprotein and carcinoembryonic antigen [85, 86]. In contrast, several, mostly immunohistochemical but also serum tumor markers have been suggested in veterinary medicine but are in most cases not routinely used in the prediction of disease outcome mostly due to low specificity and sensitivity (reviewed in [87]).

The complete sequencing of the canine, feline, bovine, equine, and chicken genome, the availability of manufactured genome microarrays, and the reduction in costs of transcriptomics and proteomics technology will however accelerate the search biomarker in veterinary sciences, and progress in this filed can be expected in the near future [88–92]. However, biomarker identification is, despite the technological progress, still difficult and requires structured and expansive analysis and comprehensive evaluation of the results.

The first and in most cases a tremendous hurdle for biomarker studies in veterinary medicine is the need for well-preserved, clinical samples with comprehensive clinical data on disease outcome. The appropriate number of samples from a well-defined study population taken with a standard protocol usually requires multicentred efforts to gain the critical mass. Once a sufficient and statistically relevant number of clinical samples are available, biomarker discovery is usually based on a simple comparison of tissue samples from patients with and without the disease (diagnostic biomarkers), certain disease outcomes (prognostic biomarkers), or different responses to therapy (stratification biomarkers). Most biomarker studies are therefore based on the assumption that specific proteins are present in the blood of a patient with a particular disease, disease outcome, or reaction to a drug. However, knowledge on the composition and the biodynamics of the blood proteome or specific proteins in healthy individuals is still far incomplete, and unless these gaps of knowledge are filled, important biomarkers may be possibly missed [93].

Whether proteins, DNA, miRNA, mRNA, or other biomolecules are searched as candidates for biomarkers in these samples depends on the available samples the concurrent knowledge on disease mechanisms, and the general requirements of the intended final assay. Nonetheless, there is a clear trend towards proteomic approaches on easily accessible body fluids like serum to identify biomarkers for clinical routine diagnostics. This is supported by the growing content of the public protein data bases (SwissProt, UniProt, RCSB) for human, murine but also other farm and companion species which facilitates the first biomarker discovery studies in veterinary science.

The work plan for biomarker discovery projects is long and complicated (reviewed in [93]). Briefly, the early detection research network (EDRN) has proposed a five-phase process for the development and testing of disease biomarkers [94].

Explorative preclinical, proteome or transcriptome studies are proposed as initial first phase steps in biomarker discovery. Which of the “-omics” strategies is utilized is in most circumstances determined by the available equipment and the amount of preexisting knowledge of molecular disease mechanisms (reviewed in [84]). Nevertheless, mass-spectrometry-based profiling by MudPIT proteomics of low molecular weight proteins (peptidome) in the clinical serum samples has been given special attention due to the easy and microinvasive obtainment of such samples in a clinical environment [95]. In contrast, the cancer biomarker-family approach is based on the hypotheses that “if a member of protein family is a biomarker, other members of that family might also be good biomarkers” [84]. Finally, the secreted-protein-based approach hypothesizes that a good serological biomarker should be a secreted protein, and biological fluids with contact to the tumor site may help in primary identification of these markers [84, 93].

The initial preclinical, explorative phase of the biomarker discovery which includes proteome or transcriptome profiling of the complete set of proteins has to be followed by the development of clinical assays for the identified few candidates. The assays are tested retrospective longitudinally and prospectively to evaluate their sensitivity and specificity for disease detection in phases two to four [94]. Finally, in phase five, the established assays have to be evaluated in a large patient population to ensure their clinical efficiency and impact, another phase which requires multicenter collaboration to obtain sufficient patient numbers [84, 93]. All of these stages are influenced by several variables that have been carefully planed or standardized by standard operating procedures but are beyond the scope of this review (reviewed in [93]).

Costs are another critical factor of biomarker discovery at each of the discovery phases. In the initial exploratory phase, the high costs of modern instrumentations and reagents for proteome and transcriptome analysis are a limiting. Costs for commercial proteome analysis are dropping. In the proceeding phases, costs are mainly driven by the development and validation of marker-specific assays, for example, ELISA. The cost for the development of the assays varies but numbers of 100,000 up to 1,000,000$ have been suggested [96]. In addition, experience shows that only a small fraction of the total candidates successfully make good clinical biomarkers [96]. The high developmental costs for specific biomarker detection assays and only to a minor extent the costs for explorative “-omics” technologies are therefore considered as the current bottleneck in biomarker discovery [97, 98]. 

### 4.2. Detection of Prognostic Gene Expression Patterns by Supervised Gene Expression Profiling

Especially in tumor biology, it becomes more and more evident that for most cancer types a malignant phenotype is associated with specific, complex changes in gene expression levels and not based on the expression levels of single genes [99, 100]. This perception has changed the search for prognostic factors in oncology. In recent years, expression profiling has been used to identify complex, prognostic gene expression patterns [100–102]. Ideally, gene expression profiling focuses on protein expression levels, since mRNA or miRNA expression levels do not fully reflect the protein expression pattern and the biologic activity of a cell. However, the technological head start of mRNA assays like microarrays and next generation sequencer makes these methods superior to proteome analysis for the identification of prognostic gene sets. In addition, prognostic gene expression patterns do not have to reflect the true biologic state of the cell. Their only function is to diagnose a disease or predict a disease outcome even if they are based on biologically irrelevant, phenotypic mRNA patterns.

**Figure 5:**
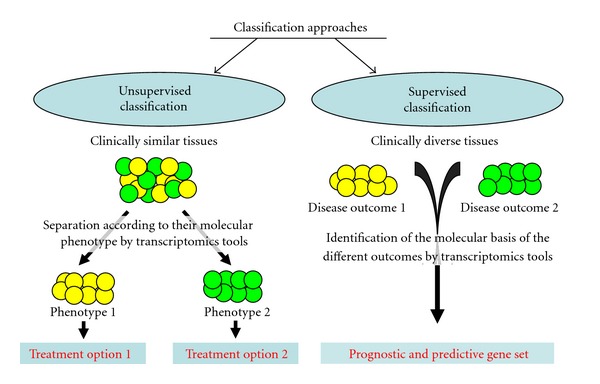
Two major approaches for the identification of disease-relevant gene expression profiles are supervised and unsupervised classification. Unsupervised classification is used to identify different molecular mechanisms in tissues from patients with clinically similar disease phenotypes. Clustering of these tissues according to their molecular phenotype may lead to the identification of different molecular phenotypes behind the identical clinical behaviour. This knowledge may finally lead to diverse therapy protocols of these molecular subtypes. In contrast, during supervised clustering, molecular mechanisms behind different disease outcome or phenotypes are analyzed. Two tissue groups with clinical differences are therefore compared, and the differences in gene expression are thought to be relevant for the distinct clinical behaviour. The resulting set of genes may also serve prognostic markers for the prospective classification tissue samples.

Two major approaches for gene identification are commonly used, supervised and unsupervised classification (Figure 5) [103]. The principle of supervised gene expression profiling studies is rather simple [99]. According to the diagnostic questions, the complete mRNA of tissue samples from “training” groups of patients with and without the disease or the intended or not intended therapy outcome are analyzed to identify genes whose expression level is significantly associated with the respective group [99]. Thus, the tissue samples are first divided by their disease status, and the data analysis aims at identifying the differences in the gene expression profile behind these clinically observed differences [99]. In contrast, the unsupervised method differentiates tissue samples according to their gene expression profile without preceding information on their diseases status. This approach aims at identifying molecular phenotypes within a by then phenotypically homogeneous disease which may be of interest for treatment and prognosis. Another method is to apply experimentally obtained information on gene expression profiles associated with the activation of certain molecular pathways to classify tumour samples. This hypothesis-driven gene expression profiling approach aims at differentiating tumours that depend on these molecular pathways and may be treated accordingly [99].

The diagnostic value of the gene sets identified with supervised clustering has to be tested in a second, independent “test” group to confirm its usefulness. This group again consists of a large population of patients with and without the disease or the different therapeutic outcomes. In an unsupervised approach, the prognostic gene set is now used to assign tissue samples to one of the groups. According to the ability to attribute clinical samples to the respective group, that is, false negative and false positive results, the specificity and sensitivity of the gene set are determined.

**Figure 6:**
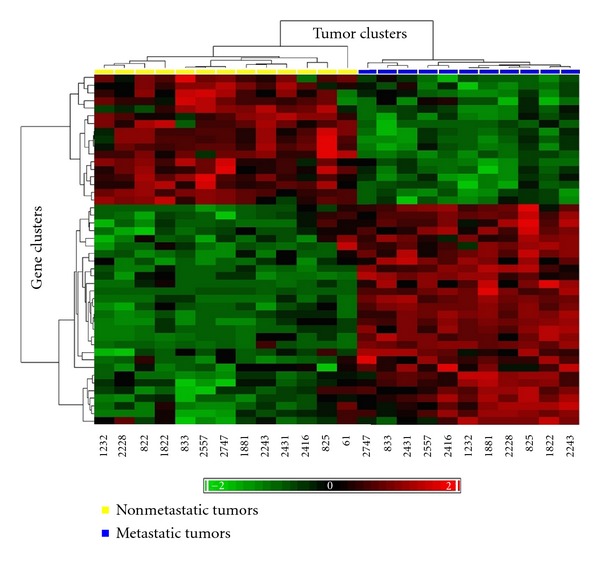
First studies in canine mammary tumours show that clinically different but histologically similar tumours can be clustered in a supervised approach into clinically relevant groups by a prognostically relevant gene sets (modified from [102]).

In 2002, van't Veer et al. published one of the first studies that identified a prognostic set of 70 genes for human breast cancer [100]. In a supervised classification approach, they compared lymph-node-negative breast cancer samples with short intervals to distant metastases with samples with long intervals to distant metastasis. By this approach, the authors identified a prognostic set of 70 genes that predicts distant metastases and allows a patient-tailored therapy strategy to identify patients which benefit from the application of adjuvant therapy from [100]. Of interest, first gene expression profiling studies on canine mammary tumours show that similar prognostic gene set may exist for malignant canine mammary tumours although their prognostic relevance has not been tested in larger patient populations yet (Figure 6) [101, 102, 104–107]. It can however be assumed that similar prognostic gene sets will be identified for veterinary diseases in the near future, given that affordable molecular tools like microarrays are now available for the most relevant species in veterinary science.

Unsupervised cluster analysis and hypothesis-driven gene expression profiling are less successful and promising strategies to identify prognostic gene expression profiles but may help to understand disease-associated molecular mechanisms in explorative and hypothesis-driven transcriptome and proteome studies. 

### 4.3. Explorative and Hypothesis-Driven Transcriptome and Proteome Analysis

Ideally, scientific progress is a continuous hypothesis-driven process by which new ideas are based on and refine recent findings. However, occasionally the preexisting knowledge is insufficient to establish a substantial hypothesis. In these cases, transcriptome and proteome analyses are a promising, explorative approach to obtain first exploitable information on potential molecular disease mechanisms. Both supervised and unsupervised gene expression clustering using microarray technology and comparative proteome analysis have been used in veterinary science to explore the molecular basis of various, mostly neoplastic diseases [64, 104, 105, 108–110]. All these studies compared diseased and nondiseased tissues in a supervised approach to identify genes or molecular pathways with significant disease association. These disease-associated proteins are now to be tested in further hypothesis-driven experiments to prove their relevance as primary disease causes or to identify them as pure phenotypic and secondary effects.

Unsupervised clustering of microarrays data has been used in human oncology to subclassify histologically identical cancer samples by their molecular phenotype. This strategy is based on the general hypothesis that a clinically and morphologically uniform disease may be caused by different molecular mechanisms, that is, that different molecular mechanisms can lead to the same disease or clinical picture. In one of the first studies using this strategy, Perou et al. investigated the gene expression patterns in breast cancer by unsupervised hierarchical cluster analysis [111]. They were able to divide malignant high-grade breast cancer tissue samples into four diverse subgroups with differential gene expression profiles. In several ensuing studies, these subgroups were refined, and their prognostic and therapeutic relevance has been confirmed [112]. Similar studies with impact on the classification of veterinary tumours are lacking but hopefully will be available in the near future.

Hypothesis-driven gene expression profiling is the complete opposite strategy to unsupervised, explorative gene expression profiling. This strategy uses gene expression signatures which either have been identified during in vitro or in vivo experiments using models of the disease of interest or theoretically by comparison with other diseases, species, or consideration of known gene functions [99]. The relevant set of genes or rather “functional” gene expression profiles are applied on preexisting microarray data to subcategorize the tissues according to the activity of the relevant genes. For instance, it has been hypothesized that carcinogenesis is a chronic inflammatory process, and a tumor can therefore be considered as nonhealing wound [99, 113, 114]. Based on this idea, the gene expression profiles of serum-activated fibroblasts were applied to the gene expression profiles of different tumours [113]. It has been shown that signature was an independent prognostic factor and showed a strong prediction of metastasis and overall survival and may therefore represent a hypothesis-based prognostic gene set [115].

Hypothesis-driven gene expression profiling studies on veterinary diseases are not available momentarily, most probably to the delayed accumulation of necessary gene expression data in experimental models. However, due to the successful application of this approach on human diseases, it can be expected that similar studies will be available in the near future. 

## 5. Summary

Transcriptome and proteome analyses have been introduced as a research tool in most fields of biomedical research. They permit the identification of prognostically relevant biomarkers, gene expression profiles, and the understanding of complex molecular mechanisms in cell physiology and pathology.

Methods to analyze the transcriptome have made the greatest advancement so far. cDNA microarrays have been and are widely used to analyse disease mechanisms and to identify prognostic gene sets. With the availability of species-specific, commercially available and affordable microarrays, it is expectable that its use is becoming even more relevant in veterinary science in the next years. However, due to the intrinsic cross-hybridization and sensitivity issues of the microarray technology, there are tendencies towards Ref-Seq or deep sequencing technologies, respectively, as an alternative method for the analysis of the transcriptome. Ref-Seq expands transcriptome analysis by the parallel information on the mutational status of the quantified mRNA types and is less dependent on the full genome coverage of a species in a database. Especially, the latter fact indicates that Ref-Seq will be helpful in areas of veterinary science with deal with rather rare species, where microarrays are commercially not available. A major but most probably temporary disadvantage of Ref-Seq is the relatively high costs and the need of computational power and biostatistical expertise required to appraise the resulting data masses. The questionable relevance of mRNA expression levels on the biology of a tissue is, however, a general problem of transcriptome analyses, independent from the applied technology.

Proteome analysis is therefore considered the ultimate method for the analysis of disease-associated mechanisms. Momentarily, the two competing methodical approaches are 2D-gel electrophoresis-based proteomics and shotgun proteomics. While 2D-gel electrophoresis allows protein separation with less expansive equipment and experience, chromatography-based proteomics seems to be of greater development potential in terms of mechanisation and standardization. General hurdles of proteome analyses itself that still need further development are problems to standardize the analysis of chemically and biochemically highly diverse proteins when in tissue lysates, that is, to cover the full proteome in each case. Furthermore, there are still sensitivity issues in mixtures of high-and low-abundance proteins and the general instability of proteins in tissue samples.

Biomarker discovery is a constantly growing scientific field which attracted high investments and efforts in the recent years. Although it can be based on all kinds of biologic elements, that is, RNA, metabolites, it is commonly based on proteome analysis of tissue samples or body fluids. Despite the initial euphoria, the revenues of the enormous investments in biomarker discovery are moderate, scientifically and commercially. In addition, biomarker discovery in veterinary science is hampered by lack of appropriate tissue banks with proper clinical data, the high costs for initial proteome analysis, and the high costs for the establishment and evaluation of subsequent diagnostic tests.

In terms of data analyses, supervised and unsupervised clustering analysis are the dominating approaches to identify disease-associated gene expression. Supervised clustering allows the identification of prognostic gene sets that may predict disease and treatment outcome and has already been successfully applied in veterinary science. It is based on the comparison of gene expression profiles of tissue samples with a known disease outcome. In contrast, unsupervised clustering is used to elucidate molecular mechanisms behind morphologically and clinically similar diseases. Application of this approach on veterinary diseases will certainly refine or even change our perception of many diseases. Hypothesis-driven gene expression profiling is a less frequently used but promising approach which experimentally derived expression profiles to identify molecular pathways with relevance in clinical tissue samples. 

In summary, “-omics” technology has made it to a standard method in human and to a minor extend also in veterinary biomedical research. They are useful tools for the explorative studies and allow the analysis of complex gene expression patterns. Given that the costs for these methods will further drop, it can be expected that the results of numerous transcriptomics and proteomics studies will contribute to the understanding of several veterinary diseases in the near future. 

## Figures and Tables

**Table 1:** Advantages and limitations of transcriptomics technologies.

	qPCR arrays	Microarrays	Ref-Seq
Advantage	Highest sensitivity/specificity	Costume-made assays available less expensive than Ref-Seq	Combination of quantitative and sequence information

Limitation	Only few hundreds of targets measurable in parallel	Less sensitive and specific than qPCR	Less sensitive and specific than qPCR Expansive

**Table 2:** Advantages and limitations of the two major proteomics approaches.

	2D-DIGE	Shotgun Proteomics (MudPit)
Advantage	Moderate costs for necessary equipment (separation phase) Good protein separation	Industrialized work processes Almost all protein types analyzable

Limitation	Gel-based variability Hydrophobic (membrane) proteins difficult to measure	Difficult quantification of low abundance proteins in complex protein mixes High costs for equipment
